# Expression of Potential Resistance Genes to the English Grain Aphid, *Sitobion avenae*, in Wheat, *Triticum aestivum*

**DOI:** 10.1673/031.013.9001

**Published:** 2013-09-22

**Authors:** Chun-Ping Wang, Zheng-Hong Wang, Hui-Yan Zhao, Qi-Di Zhu, Kun Luo, Li-Ming Wang, Pu-Hui Dong

**Affiliations:** 1 College of Agronomy, Henan University of Science and Technology, Luoyang, Henan 471003, P. R. China; 2 State Key Laboratory of Crop Stress Biology in Arid Areas / Northwest A&F University, Yangling, Shaanxi 712100, P. R. China

**Keywords:** amplified bands, F_3_ resistant populations, patterns of differential expression

## Abstract

The English grain aphid, *Sitobion avenae* (F.) (Homoptera: Aphididae), is a dominant and destructive pest in wheat, *Triticum estivum* L. (Poales: Poaceae), production regions in China and other grain-growing areas worldwide. Patterns of gene expression of the *S. avenae-resistant* synthetic wheat line 98-10-35, the *S. avenae*-susceptible line1376, and their hybrid population, and the differences in segments between 98-10-35/1376 F_3_ resistant plants and resistant parents of 98-10-35, as well as those between the F_3_ resistant and susceptible populations, were examined with differential display reverse transcription PCR. The results showed that five patterns of differential expression were detected between the progeny and its resistant parents: 1) The gene was silenced in one of the parents; 2) Special expression showed in the progeny; 3) Expression was consistent with the resistant parents; 4) Up expression showed in the progeny but not in the parents; 5) Down expression showed in the progeny but not in the parents. Paired *t-*test results were not significant; however, the probability value (0.9158) indicated that gene expression on the RNA level were consistent with resistant bands found in F_3_ resistant individuals and resistant parents, as well as the F_3_ resistant and susceptible populations. For both the F_3_ of 98-10-35/1376 and the parents, the total number of amplified bands was 202, with an average of 25.3 per primer. The number of differential bands was 116, with an average of 14.5 per primer amplified and a polymorphism ratio of 56.3%. In the present study, differential expression genes in the resistant line 98-10-35 were all up-regulated. Among them, gene expression of resistant groups in the F_3_ population was in agreement with patterns 2, 3, and 4. However, the susceptible line 1376 did not have this gene expression on the RNA level. This pattern is expected to be used to select and analyze target genes from the same F_3_ population and the resistant parents. The results suggest that it can be employed as a new method for molecular assisted breeding.

## Introduction

The English grain aphid, *Sitobion avenae* (F.) (Homoptera: Aphididae), is an important destructive pest in wheat, *Triticum estivum* L. (Poales: Poaceae), production regions in China and the world (Alkhedir et al. 2010; Razmjou et al. 2011; Liu et al. 2012). In China, the influence of & *avenae* is spread over an area of about 16.7 million hm^2^ in wheat production areas, especially in the Yellow River and Huai River basins, the North China Plain, and the southwest, northwest, and middle Yangtze River basins in China (Zhang et al. 2009). As a result, *S. avenae* infestation has caused reductions in wheat grain yield by as much as 42% (George and Gair 1979; Xu et al. 1998). Chemical control has been used to try to solve this problem, but it has led to a severe environmental pollution (Flickinger et al. 1991; Daily et al. 1998). However, efficient, large-scale control measures are not available at present. Alternatively, it has been shown that plant resistance is the most common approach in integrated pest management programs to counter the effects of *S. avenae* (Razmjou et al. 2011).

In recent years, the effect of wheat variety on major population parameters of *S. avenae*susceptible or *S. avenae-resistant* wheat varieties have been extensively investigated by numerous scientists worldwide (Özder 2002; Delp et al. 2009; Dogimont et al. 2010). Hu et al. (2004) studied German and Chinese wheat cultivars to determine their physical resistance locus to *S. avenae* by using an electrical penetration graph technique. Previous studies typically focused on the mechanism of differential expression levels of inducible resistance to wheat germplasm (Zhu et al. 2005; Dedryver et al. 2010). Some differential expression genes were obtained from these studies, but the results could not fully explain the mechanism of resistance of wheat grain to aphids (Zhu et al. 2005; Zhao et al. 2009; Zhang et al. 2012). Despite the progress in this area, the exact molecular mechanisms of the differential expressions of wheat genetic resistance to *S. avenae* require further study. Differential expression of constitutive resistance of wheat varieties to *S. avenae* is still unknown.

It has been suggested that breeding and cultivating aphid-resistant germplasm is the most economical, safe, and effective way to manage *S. avenae* infestation. Breeding plants that are resistant to *S. avenae* is considered an important method for the integrated pest management. In recent years, many effective resistance genes have been isolated with the rapid development of biotechnology, which enables molecular-assisted breeding of resistant wheat to be used in integrated pest management.

Differential display reverse transcription PCR (DDRT-PCR) has been widely used in studies of signal transduction and morphological development of plants and in identifying and cloning plant resistance genes associated with stress and disease. Many plant genes and cDNA fragments have been obtained using this technique (Xiong et al. 2002; Chen et al. 2006; Delp et al. 2009). This technique shows potential for studying the functional genomics in plants. However, no study on the differential expression resistance-related genes in wheat parents, the F_3_ generation, or the backcross populations has been reported. In addition, the expression patterns between the F_3_ resistant populations and parents or sensitive plants have not been examined.

The wheat line 98-10-35 showed resistance to *S. avenae* (Du et al. 1999; Hu et al. 2011; Wang et al. 2011). The resistance was controlled by a single dominant gene (Wang et al. 2011). The *S. avenae*-resistant line 98-10-35, the *S. avenae*-susceptible line 1376, the F_3_ generation, and the backcross population of 98-10-35/1376 were investigated in this study. The objective of this study was to examine the expression pattern of the F_3_ resistant populations, the differences in expression between parents and F_3_ resistant populations, and the differences between the sensitive and resistant plants. Expression patterns could provide an identification method for selecting target genes from candidate genes.

## Materials and Methods

### Wheat lines

The wheat lines used in this study were 98-10-35, 1376, Amigo, and resistant and susceptible plants 98-10-35/1376 F_3_ (177, 178) BC1 and BC2 population. All the wheat materials were provided by Henan University of Science and Technology, Northwest A & F University, China.

### Field trials

Materials were planted on the experimental farm of Henan University of Science and Technology. The field trials were investigated over two successive seasons (2010/2011 and 2011/2012). Each wheat germplasm line was sowed on two 100 cm long rows, 24 cm apart. Two replications were established with a completely randomized design. Guard rows were set, and susceptible line 1376 was planted on them. No insecticide was applied during the entire growing season.

### Identification of resistance to aphids

Resistance identification was conducted under the natural infection condition. For each germplasm line, the number of *S. avenae* on the 10 most seriously injured stems was counted to investigate the total number of *S. avenae* (four replicates for each germplasm) during the jointing stage and grain-filling stage. Infested plants were examined every 7 days. The wheat grain aphid index (average number of a certain germplasm of *S. avenae* per plant/average number all germplasm of *S. avenae* per plant) was calculated. The wheat grain aphid index was summarized in seven scales, as shown in Table 1 (Painter et al. 1958).

### Primers of DDRT-PCR

Primers in this study were synthesized by Sangon Biotech Co. (www.life-biotech.com).

**Table 1.**
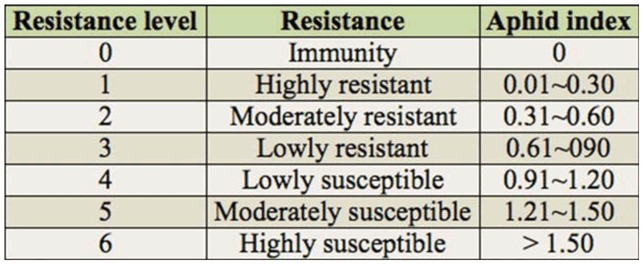
The index of what germplasm resistance to *Sitobion avenae*.

**Table 2.**
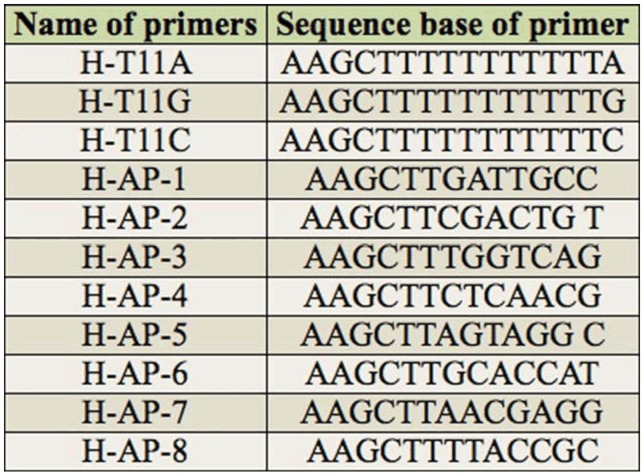
The primers used in DDRT-PCR analysis (Lao 2009).

**Figure 1.**
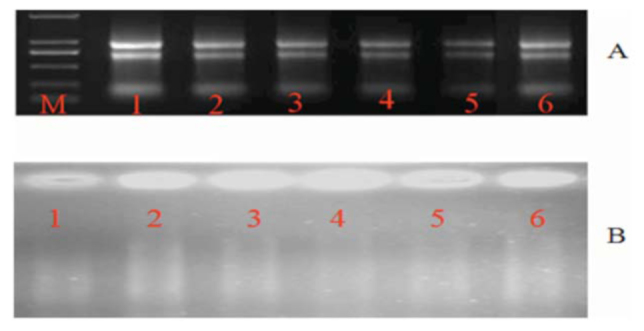
The quality testing of total RNA (A) and the firststrand cDNA (B). 1–6 are resistant and susceptible parent plants and resistant and susceptible plants of F_3_ population and BC1 population, respectively. M represents the DNA marker. High quality figures are available online.

The primers consisted of three anchor primers and eight random primers, as shown in Table 2 (Lao 2009). Meanwhile, the house-keeping gene 18srRNA primers were used to examine the quality of total RNA and successfully reversed transcription. The sequences were 5′CGTCCCTGCCCTTTGTACAC-3′ and 5′CTCC ATGTC ATCCC AGTTG-3′.

### Synthesis of the first strand of cDNA

For RNA extraction, 1 cm leaf samples were immediately submerged in 2 mL Trizol (Invitrogen, www.lifetechnologies.com), homogenized for 1 min with a tissue homogenizer, and stored at -80° C until further process (Tiangen Biotech Co. www.tiangen.com). The total RNAs were reversely transcribed to form the first chain of cDNA by PrimeScript RT Reagent Kit (TaKara Biotechnology Co., www.clontech.com/takara). Quality testing of RNA extraction and first strand cDNA are shown in Figure 1.

### DDRT-PCR

The 15 µL reaction mixtures consisted of 1.5 µL reaction products of the first chain of cDNA, 1.5 µL Mg-free 10×PCR buffer, 1.5 unit of Taq DNA polymerase, 1.3 µL 2.5 mM OfMgCl_2_, 1.3 µL 2.5 mM of dNTP, and 1.0 µL 10 µL of each primer pair and ddH_2_O. After 5 min of denaturation at 94° C, amplifications were conducted for 25 cycles, with each cycle consisting of denaturing 30 sec at 94° C, annealing 1 min at 45° C, and extension 1 min at 72° C followed by 15 cycles, each one consisting of 30 sec at 94° C, 1 min at 50° C, 1 min at 72° C, followed by extension at 72° C for 10 min.

### Data Analysis

A matching *t*-test was done with SPSS 13.0 (IBM, www.ibm.com). The amplified bands were analyzed with Quantity One software (Bio-Rad, www.bio-rad.com). The stable bands were recorded in two repeat amplifications from 50 bp to 1000 bp. The lane with a detected band was marked ‘1’, otherwise it was marked ‘0’.

## Results

### Identification for resistance to aphid

The results showed that wheat line 1376 was a highly susceptible line, and 98-10-35 and Amigo were moderately resistant lines. The BC1 and F_3_ populations were segregated in two parts, including moderately resistant and highly susceptible. Average aphid indexes of resistant and susceptible plants are listed in Table 3.

**Table 3.**
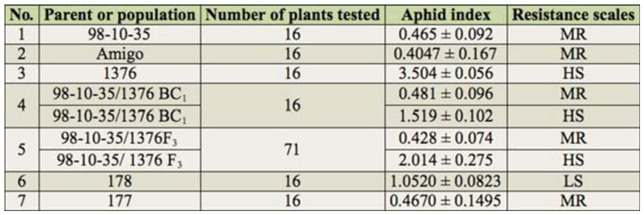
Resistance evaluation of parent or population to *Sitobion avenae*.

**Figure 2.**
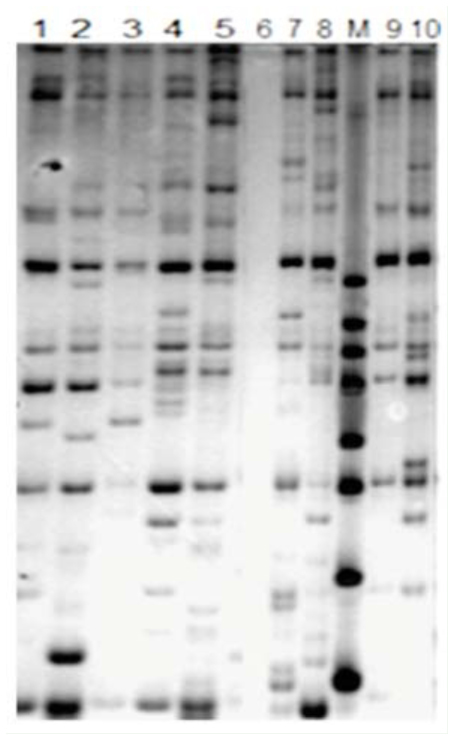
8% non-denatured PAGE picture of differential results. 1–10 and M represent two F_3_ plants of Amigo/1376, 177 line, 178 line, F_3_ plant of 98-10-35/1376, 1376, Amigo, 98– 10–35, BC1 plant of 98–1035/1376, BC_2_ plant of 98–10– 35/1376, and marker, respectively. High quality figures are available online.

**Figure 3.**
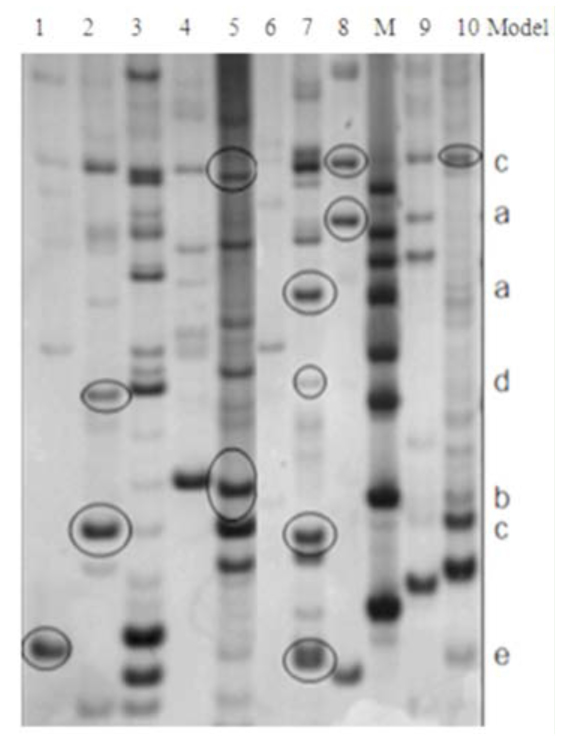
Expression patterns of hybrids and their models in wheat. High quality figures are available online.

**Figure 4.**
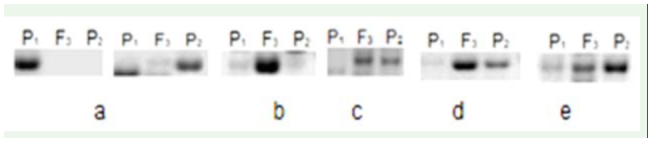
Gene expression models of parent and its F_3_. Each type of three bands from left to right represents male parent 1376, F_3_ plant of 98-10-35/1376, and female parent 98-10-35, respectively. High quality figures are available online.

### Analysis of the differential transcriptderived fragments

Three anchor primers and eight random primers consisted of 24 DDRT-PCR primer pairs, which were applied to test all plants in order to find the differential expression transcript-derived fragments (TDF) (Figure 2).

### Differences of expression patterns in revealing a candidate gene

Twenty-four pairs of primers were employed to conduct the repeating test to screen wheat resistance genes to *S. avenae* in all samples. The results showed that five patterns of differential expression were detected between the progeny and its resistant parents (Figure 3): (1) The gene was silenced in one of the parents (a); (2) One of the parents appeared in bands, while others had no bands. Special expression showed in progeny (b); (3) The gene only occurred in hybrid offspring bands, and two parents had no bands. Express consistency showed with resistant parents (c), which means that bands for resistant parents and offspring appeared, but did not appear in the sensitive parents; (4) Up-expression only showed in progeny and not in either parent (d); (5) Both the parents and offspring expressed the resistance bands, but the amount of expression in the offspring was much more than the parents. Down expression only showed in progeny and not in either parent (e).

According to the amplification, five kinds of differential expression patterns can be detected in the samples. Analysis and comparison of the amplified bands showed that there were slight differences among the F_3_ generation, backcross offspring of 98-10-35/1376, and the parents. In the genetic process, its expression model mainly appeared in five patterns, as shown in Figure 4.

**Table 4.**
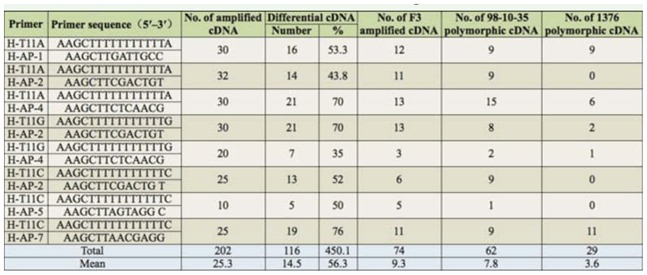
Results of TDFs of candidate genes.

**Table 5.**
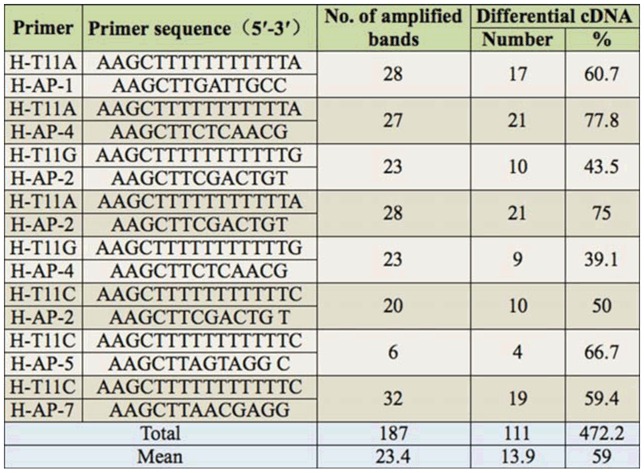
Results of differential fragments of candidate genes.

### Expression differences analysis of TDFs in F_3_ of 98-10-35/1376 and parents

TDF groups for F_3_ of 98-10-35/1376 and parents were amplified via eight pairs of differential DDRT-PCR primer. Analysis and comparison of amplification figures showed some differences in TDFs among the F_3_ of 98– 10–35/1376, backcross offspring, and parents.

TDFs changed to a certain extent in type and number on F_3_ of 98-10-35/1376 and parents, with the statistics segment size being 50–1000 bp. Statistical results of differentially expressed fragments are shown in Table 4.

The results showed that the total number of amplified bands was 202 for the F_3_ of 98–10– 35/1376 and parents, with an average of 25.3 bands per primer pair. There were 116 differential bands, with an average of 14.5 bands per primer pair and a polymorphism ratio of 56.3%. From the analysis and comparison ofmutual bands on F_3_ of 98-10-35/1376 and parents, there were 74 differentially expressed bands for F_3_ generation of 98-10-35/1376, with an average of 9.3 bands per primer. There were 62 differentially expressed bands for the *S. avenae*-resistant line 98-10-35, with an average of 7.8 bands per primer. There were 29 differentially expressed bands in 1376, with an average of 3.6 bands per primer. The fragments matching *t*-test showed that *p* = 0.9158 and showed no difference for the F_3_ generation of 98-10-35/1376 and 98-10-35 differentially expressed. The F_3_ of 98–10– 35/1376, the line 98-10-35, and line 1376 exhibited significant differences in the amplified products, i.e., gene expression products only existed in the F_3_ generation.

### Different TDF displays of F_3_ generation resistant and sensitive single plants

TDF groups for resistant single plants and sensitive single plants of F_3_ of 98-10-35/1376 were amplified by using eight pairs of differential primers with a TDF segment size of 50–1000 bp.

The types and numbers of TDFs of the resistant single plants and sensitive single plants varied greatly on the F_3_ of 98-10-35/1376. The results showed that the total number of bands amplified was 187 in the resistant single plant group and sensitive single plant of F_3_ of 98– 10–35/1376, with an average of 23.4 bands per primer (Table 5). There were 111 differential bands, with an average of 13.9 bands per primer amplification and a polymorphism ratio of 59%. The differential expression gene products appeared in the F_3_ of resistant and sensitive single plants. The resistant single plants and sensitive single plants of the F_3_ population of 98-10-35/1376 showed differences in amplified products when different primers were used.

In summary, differential expression genes in resistant line 98-10-35 were all up-expressed. The gene expression of resistant groups in the F_3_ generation was in agreement with the patterns 2, 3, and 4 (Figure 2). The sensitive cultivar 1376 failed to express.

## Discussion

### DDRT-PCR

At present, there are several methods for identifying differentially expressed sequence tags, such as northern blotting, differential screening, microarray analysis, subtractive hybridization, serial analysis of gene expression, cDNA amplified fragment length polymorphism, and DDRT-PCR (Lao 2009). This study focused on the molecular mechanism of wheat germplasm resistance to *S. avenae*. From the aspect of gene expression, differential expression was explored by breeding resistant cultivars to aphid, and some of available genes were obtained by using DDRT-PCR.

DDRT-PCR is a cDNA differential display technique that combines RNA reverse transcription with traditional PCR technology. This technique is highly sensitive and has a wide range of applications, such as testing the differential expression of genes at the cellular level (Xiong et al. 2002; Chen et al.2006; Delp et al.2009). It is an important differential expression technology and can be widely used to understand gene expression characteristics of organisms. Recently, DDRT-PCR has been successfully used to isolate expressed sequence tags in a variety of organs. In this study, candidate genes of resistance to *S. avenae* were reported for the first time by using this method. A new method of studying genetic resistance breeding by aiming at the same gene in all samples was proposed.

### Differential expression pattern of candidate genes of resistance to aphids

Delp et al. (2009) reported that protease inhibitors serine/threonine kinase exist in constitutive resistance of soybean cultivars resistant to *Rhopalosiphum padi*. This result implies that there should be some resistant genes in the resistant soybean cultivars such as 98-10-35. In this study, five kinds of differentially expressed patterns were described in the parents, F_3_ population, and backcross population, which is a slightly different result compared to previous studies on cotton, wheat, and barley (Xing et al 2005; Meng et al 2005; Zhang et al 2008; Zhu et al 2009; Zhang et al 2010). Four differential expression models were detected between the hybrid and its parents in cotton: (1) Up expression only showed in hybrid and not in either parent; (2) Dominant expression showed in one of the parents but not in F1 and another parent, including the expression pattern in the female parent and the hybrid but not in the male parent, and the expression pattern in the male parent and the hybrid but not in the female parent; (3) The gene was silenced in one of the parents, including the expression pattern in the male parent but not in hybrid and female parent, and the expression pattern in the female parent but not in the hybrid and the male parent; (4) Down expression showed in both parents but not in the F_1_ generation (Zhu et al 2009). Previous studies also showed four differential expression models. In the present study, five expression patterns were found in the expression of resistant genes to *S. avenae*. At the same time, different bands in gene expression were shown between parents and F_3_ resistant populations and between the sensitive and resistant plants. It is best to select the target genes among lots of candidate genes by choosing from three patterns (2, 3, and 4 type) in the present study in order to save labor force and financial resources and overcome the blindness of selection. This study is of significance for research on resistance of wheat to *S. avenae* and suggests a new approach to study genetic resistance breeding.

## Abbreviations:

DDRT-PCR,: differential display reverse transcription PCR;
TDF,: transcript-derived fragments

## References

[bibr01] Alkhedir H, Karlovsky P, Vidal S (2010). Effect of light intensity on colour morph formation and performance of the grain aphid *Sitobion avenae* F. (Homoptera: Aphididae).. *Journal of Insect Physiology*.

[bibr02] Botha WJ, Jaftha JB, Bloem JF, Habig JH, Law IJ (2004). Effect of soil bradyrhizobia on the success of soybean inoculant strain CB 1809.. *Microbiological Research*.

[bibr03] Botha AM, Lacock L, Niekerk C, Matsioloko MT, Preez FB, Loots S, Venter E, Kunert KJ, Cullis CA (2006). Is photosynthetic transcriptional regulation in *Triticum aestivum* L. cv. ‘TugelaDN’ a contributing factor for tolerance to *Diuraphis noxia* (Homoptera: Aphididae)?. *Plant Cell Reports*.

[bibr04] Botha AM, Li Y, Lapitan NLV (2006). Cereal host interactions with Russian wheat aphid: a review.. *Journal of Plant Interactions*.

[bibr05] Chen YH, Zhao S, Yan QQ, Li YS, Xiao GY (2006). Studies on Genes Related to Submergence Tolerant Using Differential Display Technique in Rice.. *Journal of Agricultural Biotechnology*.

[bibr06] Cao YZ, Yin J, Li KB, Zhang KB, Li XQ (2006). Exploration of the factors causing the outbreak of the wheat grain aphid and the control strategies.. *Plant Protection*.

[bibr07] Daily G, Dasgupta P, Bolin B, Crosson P, du Guerny J, Ehrlich P, Folke C, Jansson AM, Jansson BO, Kautsky N, Kinzig A, Levin S, Maler KG, Pinstrup-Andersen P, Siniscalco D, Walker B (1998). Policy forum: Global food supply: food production, population growth, and the environment.. *Science*.

[bibr08] Dedryver CA, Le Ralec A, Fabre F (2010). The conflicting relationships between the wheat grain aphid and men: A review of aphid damage and control strategies.. *Comptes Rendus Biologies*.

[bibr09] Delp G, Gradin T, Ahman I, Jonsson LMV (2009). Microarray analysis of the interaction between the aphid *Rhopalosiphum padi* and host plants reveals both differences and similarities between susceptible and partially resistant barley lines.. *Molecular Genetics and Genomics*.

[bibr10] Dogimont C, Bendahmane A, Chovelon V, Boissot N (2010). Host plant resistance to the wheat grain aphid in cultivated crops: Genetic and molecular bases, and interactions with aphid populations.. *Comptes Rendus Biologies*.

[bibr11] Du LF, Zhao HY, Yuan F, Sun Q, Zhang GS, Yao JX, Li Y, Liu HW, Wang JW (1999). Resistance to aphid determining and screening in wheat species (lines) or sources.. *Acta Botantica Boreali-Occidentalia Sinica*.

[bibr12] Flickinger EL, Juenger G, Roffe TJ, Smith MR, Irwin RJ (1991). Poisoning of Canada geese in Texas by parathion sprayed for control of Russian wheat aphid.. *Journal of Wildlife Diseases*.

[bibr13] George K, Gair R (1979). Crop loss assessment on winter wheat attacked by the grain aphid, *Sitobion avenae* (F.), 1974–77.. *Plant Pathology*.

[bibr14] Guo BH, Gao SC, Jing RL, Wang G (2003). Studies on the Differential Expression cDNA Induced by Water-Stress in Wheat Seedling Stage.. *Biotechnology Information*.

[bibr15] Huang X, Gong ZZ, Xu ZY, Tang XH (2000). Isolation of Related cDNA Clone Resistant to Blast of Rice by mRNA Differential Display.. *Jiansu Journal of Agricultural Sciences*.

[bibr16] Hu XS, Zhao HY, Heimbach U, Thieme T, Li J, Zhang YH, Liu BM, Li DH, Hu ZQ (2004). Study on cereal aphid resistance on three winter wheat cultivars introduced into China.. *Acta Botanica Boreali-Occidentalia Sinica*.

[bibr17] Hu XS, Liu XF, Hu ZQ, Zhang YH, Zhao HY, Zhang GS (2011). The resistance of 10 wheat varieties to *Sitobion avenae* (Homoptera: Aphididae) in wheat seedlings phase in lab.. *Plant Protection*.

[bibr18] Jabraeil R, Shirzad R, Bahram N, Gadir NG, Hooshang RD (2011). Resistance and susceptibility of various wheat varieties to *Sitobion avenae* (Hemiptera: Aphididae) in Iran.. *Applied Entomology Zoology*.

[bibr19] Lao QH (2009). *Gene related to wool fineness screening with DDRT-PCR*..

[bibr20] Li YJ, Li B, Liu JZ, Lij Y, Yao SJ, Li ZS (1998). Chromosomal Location of the Genes Coding for Acid Phosphatase and Alkaline Phosphatase in *Agropyron elongatum* (2n=2x=14, EE).. *Acta Genetica Sinica*.

[bibr21] Meng FR, Ni ZF, Wu LM, Xie XD, Wang ZK, Sun QX (2005). Differential Gene Expression between Reciprocal Crossfertilized Kernels and Their Parents during the Early Stages of Seed Development in Wheat.. *Acta Agronomica Sinica*.

[bibr22] Özder N (2002). Development and fecundity of *Sitobion avenae* on some wheat cultivars under laboratory conditions.. *Phytoparasitica*.

[bibr23] Painter RH (1958). Resistance of plants to insects.. *Annual Review of Entomology*.

[bibr24] Wang CP, Chen Q, Luo K, Zhao HY, Zhang GS, Tlali RM (2011). Evaluation of Resistance in Wheat Germplasm to the Aphids, *Sitobion avenae* Based on TOPSIS and Cluster Methods.. *African Journal of Agricultural Research*.

[bibr25] Wang CP, Zhao HY, Zhang GS, Luo K (2011). *Location of Sa1 genes for resistance to wheat aphid*..

[bibr26] Liu XL, Yang XF, Wang CY, Wang YJ, Zhang H, Ji WQ (2012). Molecular mapping of resistance gene to English grain aphid (*Sitobion avenae* F.) in *Triticum durum* wheat line C273.. *Theoretical Applied Genetics*.

[bibr27] Xiong YH, Xu Y (2002). Techanological Advances of Serial Analysis of Gene Expression.. *Chinese Journal of Biotechnology*.

[bibr28] Xing CZ (2005). *Genetic Ecffct of Hybrid Cotton and the Relationship Between Gene Differential Expression and Heterosis*..

[bibr29] Xu LM, Qi FM, Zhang JP, Zhang JZ, Cao CM, Chen JL (1998). Initial measurement of wheat output loss due to *Macrosiphum avenae*.. *Inner Mongolia Agricultural Science Technology*.

[bibr30] Zhang J, Ku LX, Zhang WQ, Yang S, Liu HY, Zhao RF, Chen YH (2010). QTL Mapping of Internodes Length above Upmost Ear in Maize.. *Journal of Maize Sciences*.

[bibr31] Zhang Y, Yu XD, Tang KX, Xia LQ (2012). Generation of Aphid Resistant Transgenic Wheat with aha from *Arisaema heterophyllum* by Particle Bombardment.. *Acta Agronomica Sinica*.

[bibr32] Zhang Y, Ni ZF, Yao YY, Sun QX (2008). Differential Gene Expression in Uppermost Internode between Wheat Hybrid and Its Parents.. *Acta Agronomica Sinica*.

[bibr33] Zhang YJ, Jiang YY, Feng XD, Xia B, Zeng J, Liu Y (2009). Occurring trends of major crop pests in national significances in 2009.. *China Plant Protection*.

[bibr34] Zhao L, Chen J, Cheng D, Sun J, Liu Y, Tian Z (2009). Biochemical and molecular characterizations of *Sitobion avenae* induced wheat defense responses.. *Crop Protection*.

[bibr35] Zhao DZ, Chen M, Zhong K, Tan KH (1998). An Improved Method for Constructing Hybrid Arrested cDNA Library.. *Progress In Biochemistry Biophysics*.

[bibr36] Zhu CS (2005). *Molecular mechanism of induced resistance in wheat after aphid* (*spp*.) *feeding and the changes aphid behavior*..

[bibr37] Zhu XX, Zhu YC, Ai NJ, Liu RZ, Zhang TT (2009). Gene Differential Expression at Seedling Stage in Four Cotton Combinations Hybridized by CRI-12 and Its PedigreeDerived Lines.. *Acta Agronomica Sinica*.

